# Study of Physico-Chemical Properties and Morphology of Phospholipid Composition of Indomethacin

**DOI:** 10.3390/nano12152553

**Published:** 2022-07-25

**Authors:** Elena G. Tikhonova, Yulia A. Tereshkina, Lyubov V. Kostryukova, Yulia Yu. Khudoklinova, Maxim A. Sanzhakov, Anna O. Tamarovskaya, Oleksandr I. Ivankov, Mikhail A. Kiselev

**Affiliations:** 1 Institute of Biomedical Chemistry, 10 Pogodinskaya St.,119121 Moscow, Russia; elena.tikhonova_@mail.ru (E.G.T.); burova13@gmail.com (Yu.A.T.); kostryukova87@gmail.com (L.V.K.); sanwakov@mail.ru (M.A.S.); 2 Joint Institute for Nuclear Research, 6 Joliot-Curie St., 141980 Dubna, Russia; bukstehude@mail.ru (A.O.T.); ivankov@jinr.ru (O.I.I.); kiselev@jinr.ru (M.A.K.)

**Keywords:** phospholipid nanoparticles, Indolip, vesicles, small-angle neutron scattering, lyophilization, physico-chemical properties

## Abstract

Nonsteroidal anti-inflammatory drugs (NSAIDs), inhibitors of cyclooxygenase-2, an enzyme involved in the formation of anti-inflammatory prostaglandin PGE2, are the most common treatment for chronic inflammatory diseases, such as, for example, arthritis. One of the most commonly used drugs of this class is indomethacin, a derivative of indolylacetic acid. In this work, we studied the physicochemical properties of the phospholipid composition of indomethacin obtained earlier (codenamed “Indolip”) and the effect of freeze drying on its parameters. It was shown that the properties such as particle size, light transmission, phospholipid oxidation index did not change significantly, which indicated the stability of the drug after lyophilization. Measurement of the spectra of small-angle neutron scattering has shown that morphologically, Indolip is a vesicle whose radius is five times greater than the value of the bilayer thickness.

## 1. Introduction

Nonsteroidal anti-inflammatory drugs are the most common treatment for chronic inflammatory diseases. One of the most commonly used drugs of this class is indomethacin, whose mechanism of action is based on the inhibition of cyclooxygenase-2, an enzyme involved in the formation of anti–inflammatory prostaglandin PGE2. However, treatment with indomethacin often leads to manifestation of certain side effects, including cardiovascular and gastrointestinal disorders. Therefore, there is a need to develop more advanced methods of delivering such drugs to the body, including the use of nanoparticles. The supply of medicinal compounds with transport systems reduces side effects: they reduce toxicity and increase the bioavailability of substances (especially poorly soluble in water), increase the rate of transition through natural barriers (cell membranes, blood-brain barrier, etc.), as well as sorption or body metabolism [1,2,3]. Much attention is paid to the development of nanosystems for indomethacin from various polymers and nanomaterials (polyglyceroladipate, Eudragit L100, polycaprolactone, chitosan, dextran), nanoparticles with a given solubility depending on the pH of the medium [4,5,6,7,8,9]. It should be emphasized that the main disadvantage of such systems is a relatively large particle size (200–400 nm), which makes them susceptible to lysis by the reticuloendothelial system of the cell. As a result, the effectiveness of the drug transported is significantly reduced. It is known that as a result of the use of transport systems, it is possible to change the pharmacokinetics of the drugs they carry, optimizing the processes of absorption, distribution in tissues, metabolism and elimination [10,11]. Regardless of the way a drug enters the body, the size of the transporting particles strongly affects its effectiveness—it has been shown that size has a greater effect on pharmacokinetics than other properties, for example, surface charge [12]. Another property of transport systems is their ability to protect medicinal compounds from premature degradation, thereby increasing the circulation time of the substance in the bloodstream and its bioavailability [12,13].

The medicinal composition based on plant phosphatidylcholine and anti-inflammatory drug substance indomethacin developed at the Institute of Biomedical Chemistry (IBMC, Moscow, Russia) was obtained using the technology described in the USA patent [14]. Nanoscale particles may help to overcome various physiological barriers in the body. The phospholipid-based composition makes it possible to obtain a biocompatible transport system that does not have a negative effect on healthy organs, since phospholipids are a natural component of the cell membrane. After oral administration of this phospholipid composition to rats, the increase in the bioavailability of indomethacin was twice as high compared to the free substance. Two inflammation models (adjuvant arthritis in rats and conconavalin-induced edema in mice) showed increased anti-inflammatory activity of indomethacin included into phospholipid nanoparticles compared with its free form [1,15]. Thus, it has been shown that the phospholipid transport nanosystem is capable of changing both the pharmacokinetics of a substance and its physical and chemical characteristics, for example, solubility.

It should be noted that the morphology of phospholipid nanoparticles loaded with a medicinal substance and used as a transport system has not been studied before. Such experiments are possible due to the use of small-angle neutron scattering (SANS). Previously, a polydisperse population of dimyristoylphosphatidylcholine (DMPC) vesicles in an aqueous sucrose solution was studied at the Joint Institute for Nuclear Research (Dubna) using the methods of small-angle neutron scattering (SANS) and small-angle scattering of X-ray synchrotron radiation (SAXS) [16]. The present work is aimed at studying the physicochemical properties and morphology of the phospholipid composition of indomethacin under the code name “Indolip”. This paper is the first to study the morphological features of the nanoparticles obtained, their size, electrokinetic potential (ζ-potential) and other physicochemical parameters after lyophilization of the developed phospholipid composition of indomethacin. These parameters play an important role in obtaining the desired pharmacokinetic and pharmacological properties of the developed transport systems, and their study will allow for a more detailed understanding of the mechanism of therapeutic action of medicinal compositions.

## 2. Materials and Methods

### 2.1. Materials

The following materials were used: soy phospholipid Lipoid S100 (“Lipoid”, Ludwigshafen, Germany) with a phosphatidylcholine content of ≥95%, indomethacin substance (“Huzhou Synthetic Pharmaceutical Factory”, Huzhou, China), maltose monohydrate (“Merck”, Darmstadt, Germany), rectified ethyl alcohol 96% (Konstanta-Farm M, Moscow, Russia), methanol for HPLC, acetonitrile for HPLC gradient analysis (Scientific UK Ltd., Stoke-on-Trent, UK), trifluoroacetic acid (TFA) ≥99% (Acros organics, Fair Lawn, NJ, USA), distilled or purified water (Milli-Q), a kit for enzymatic colorimetric determination of phospholipids (Sentinel Ch. SpA, Milano, Italy).

**Scheme 1:**
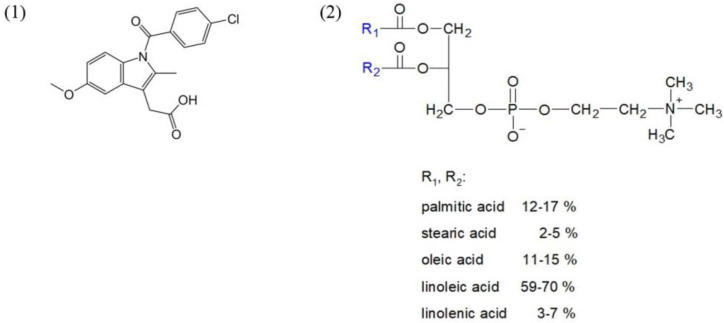
Structures of indomethacin (**1**) and phosphatidylcholine (**2**).

Indomethacin and phosphatidylcholine structures are presented below in Scheme 1.

### 2.2. Methods

#### 2.2.1. Phospholipid Nanocomposition of Indomethacin Preparation

**Figure 1:**
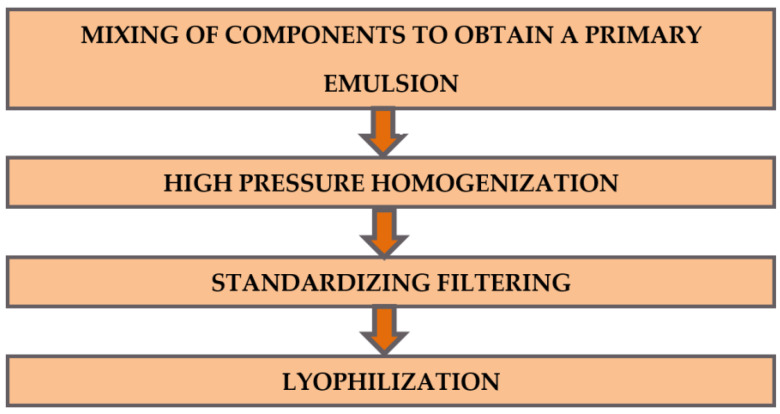
The scheme of obtaining the composition of Indolip.

The phospholipid nanocomposition of indomethacin was obtained according to the procedure described [15]: 200 mL of distilled water was warmed to 45 °C and 25 g of maltose was added with stirring until completely dissolved. Then, 625 mg of indomethacin and 6.25 g of phospholipid were added to the resulting solution, homogenized and adjusted to 250 mL with distilled water. The resulting coarse emulsion was passed through a homogenizer (Mini-Lab 7.3 VH, Rannie, Hirtshals Denmark) at a pressure of 800 bar for 5 cycles and filtered then through a filter with a pore size of 0.22 microns (Durapore, Merck, Darmstadt, Germany), poured into 10 mL vials and freeze-dried on a Virtis Advantage XL laboratory unit (Gardiner, New York, NY, USA), following [15]. Schematically, the process of obtaining the Indolip composition is shown in Figure 1.

After that, to determine the properties and study the effect of lyophilization, the dried powder was diluted with distilled water to a volume of 10 mL.

#### 2.2.2. Particle Size Determination

Particle size was determined using dynamic light scattering on a photon correlation spectrometer Zetasizer Nano ZS (Malvern, UK). Three measurements were carried out, the result in the form of polydisperse distribution of particles by volume was averaged.

#### 2.2.3. Determination of the ζ-Potential Value

ζ-potential was measured in the reduced nanoemulsion using electrophoretic light scattering on the Zetasizer Nano ZS analyzer. Three measurements were performed, the result in the form of polydisperse distribution of particles by volume was averaged.

#### 2.2.4. Determination of Light Transmission

Light transmission was determined on an Agilent 8453 spectrophotometer (Agilent Technologies, Waldbronn, Germany) using the HP UV Visible ChemStation program version A10.01 (Hewlett-Packard, Wilmington, DE, USA) at 660 nm and an optical path length of 1 cm.

#### 2.2.5. Determination of Indomethacin Content

The content of indomethacin in the composition was controlled by HPLC. The aliquot of the reduced nanoemulsion was diluted with methanol 10 times and chromatographed on an Eclipse XDB-C18 column (4.6 mm × 150 mm, 5 microns, Agilent Technologies) with a mixture of acetonitrile with 0.1% TFA and 0.1% TFA (3:2). Detection was performed at a wavelength of 254 nm (reference wavelength of 360 nm). The concentration of indomethacin in the sample was determined by the external standard method.

#### 2.2.6. Determination of Phosphatidylcholine (PC) Content

The content of PC in the samples was determined using enzymatic kits for quantitative colorimetric determination of phospholipids in serum and plasma. A sample containing phosphatidylcholine was added to a solution with enzymes. Under the action of phospholipase D, phosphatidylcholine was hydrolyzed to choline and phosphatidic acid, choline was oxidized to betaine under the action of choline oxidase, and the hydrogen peroxide formed, under the action of peroxidase, reacted with 4-aminoantipyrine and a phenol derivative, forming a red-colored compound. The color intensity proportional to the amount of phosphatidylcholine in the sample was evaluated spectrophotometrically by absorption at a wavelength of 520 nm compared with absorption at a reference wavelength of 660 nm. Measurements were carried out relative to a sample of reagents without phospholipids. To improve the accuracy of calculation, an amendment was made, determined by the ratio of the expected and determined phospholipid concentration in the standard, as which phosphatidylcholine Lipoid S100 was taken.

#### 2.2.7. Determination of Lysophosphatidylcholine (LysoPC) Content

The content of lysoPC was determined using a Beckman Altex 421 HPLC chromatograph (Brea, CA, USA) equipped with an isocratic pump and an LKB UVicord S2 UV photometric detector (LKB Bromma, Sollentuna, Sweden). The Altex Ultrasphere Si column (4.6 mm × 150 mm) was used, the mobile phase was composed of acetonitrile:methanol:water in a ratio of 216:72:12 (by volume). The flow rate of 0.5 mL/min was recorded at 206 nm.

#### 2.2.8. Determination of the Phospholipid Oxidation Index

To determine the phospholipid oxidation index, lipophilic substances were extracted from a weighed quantity of the sample using the Folch method. The extract was centrifuged at 4 °C, 10,000 rpm for 10 min (Eppendorf 5810R, rotor FA-45-30-11, Hamburg, Germany), the supernatant was dried on a rotary evaporator Laborota 4000 (Heidolph, Schwabach, Germany) at a temperature of 30–40 °C. The resulting film was dissolved in methanol and the optical density of the sample was determined on an Agilent 8453 spectrophotometer at two wavelengths of 215 and 233 nm relative to methanol. The oxidation index J was calculated by the formula: J = A_233_/A_215_, where A_233_ and A_215_ are the optical density measured at wavelengths of 233 and 215 nm, respectively.

#### 2.2.9. Determination of the Peroxide Number

Determination of the peroxide number was performed according to the generally accepted method [17]. For this purpose, lipophilic substances were pre-extracted from the sample analyzed using the Folch method due to the possible influence of maltose present in the analyzed samples on the indications.

#### 2.2.10. Measurements of the Spectra of Small-Angle Neutron Scattering

Measurements of the spectra of small-angle neutron scattering were carried out on the YuMO instrument (named in honor of its creator—Yu. M. Ostanevich) located on the IBR-2 fast neutron pulsed reactor (Dubna) [18]. Samples of the freeze-dried indomethacin composition were examined with further reduction in heavy water by adding water to the vial until the concentration of indomethacin in the solution of 5, 10 and 25% was obtained. The measurements were carried out in Hellma quartz cuvettes at temperatures of 20 and 37 °C. The spectra of small-angle neutron scattering measured on the YuMO instrument were processed using the SasView software (version 4.01) to determine the parameters of vesicular systems.

#### 2.2.11. Statistical Processing

All experiments were conducted three times. The article presents the data as an average value ± the standard error of the mean. The differences were considered significant at *p* < 0.05.

## 3. Results and Discussion

### 3.1. The Effect of Lyophilization on Physico-Chemical Properties of Phospholipid Composition of Indomethacin

**Figure 2:**
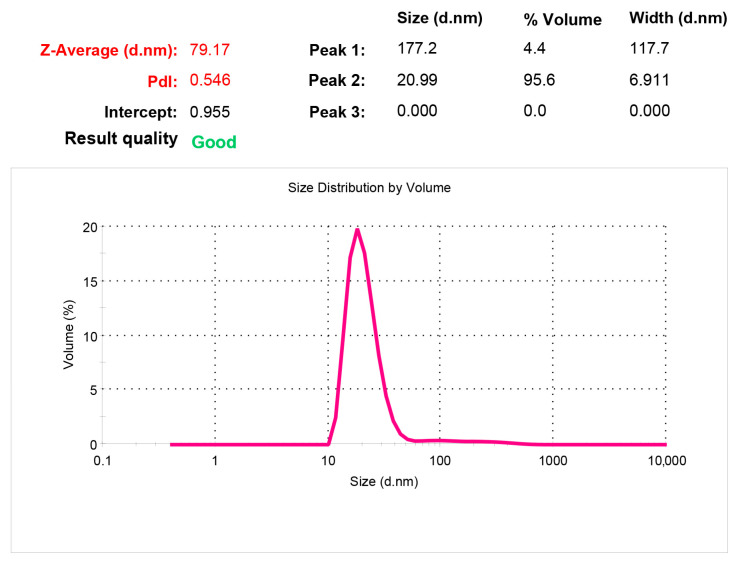
Dynamic light scattering spectrogram characterizing the polydisperse distribution of particle sizes by volume in a freeze-dried Indolip sample after its reduction in water.

The developed phospholipid emulsion with indomethacin must meet certain requirements both before and after lyophilization. Thus, in the first stage, its characteristics were studied in terms of physico-chemical parameters. After lyophilization, the indomethacin composition was a freeze-dried substance in a maltose matrix and easily soluble in water. According to the analysis, in the colloidal solution obtained after dissolution, phospholipid nanoparticles with embedded indomethacin had an average size of 21.9 ± 0.9 nm. A typical measurement spectrogram in Figure 2 shows that 95.6% of the volume occupied by all the particles in solution was accounted for by those with a size of 20.99 nm. This means that the phospholipid composition was a highly homogeneous system, so the distribution of the substance included should have been uniform.

Table 1 shows the average values of nanoparticle diameter (polymodal distribution by volume) of the main fraction of nanoparticles and light transmission before and after Indolip lyophilization.

It is obvious that the average diameter of the particles before and after lyophilization was approximately the same. Light transmission (transparency) decreased slightly after lyophilization, but its value remained above 60%. We have experimentally observed that high values of light transmission (more than 60%) at a wavelength of 660 nm in the solutions containing phospholipid nanoparticles indicate that the solutions are highly homogeneous. At the same time, the size stated in Table 1 is characteristic of particles occupying over 90% of the volume occupied by all particles present in the sample.

After determination of the ζ-potential of reduced nanoemulsion, the following value was obtained: −12.9 ± 0.6 mV. Its negative value indicates the negative surface charge of the particles forming the nanoemulsion. An absolute value of ζ-potential indicates insufficient electrostatic stabilization of nanoparticles in the solution. It is believed that the aggregation of charged particles is least likely at |ζ| > 30 mV [3]. At the same time, the freeze-dried form allows for long-term storage of the resulting composition. Moreover, our own research shows that after restoration, the nanoemulsion retains its properties within a day, and there are no signs of particle aggregation during this time.

Thus, the ζ-potential of phospholipid nanoparticles changes upon the inclusion of indomethacin. The ζ-potential of a drug-free sample of phospholipid nanoparticles is −3.9 ± 1.1 mV, i.e., with the inclusion of indomethacin, the absolute value of the ζ-potential increases to −12.9 ± 0.6 mV.

HPLC analysis showed that the average indomethacin content in the sample was 2.4 ± 2 mg/mL. The content of phosphatidylcholine, according to the enzyme-colorimetric analysis, was 24.5 ± 5 mg/mL. Thus, the mass ratio of the components of phospholipid-indomethacin (10:1) composition was experimentally confirmed.

One of the quality criteria of liposomal preparations is the oxidation of phospholipids forming a lipid bilayer. The results of spectrophotometric study, calculations of the phospholipid oxidation index in the preparation before and after freeze drying, as well as the content of lysoPC are presented in Table 2.

As can be seen from Table 2, the lyophilization process did not lead to a significant increase in the oxidation of phospholipids in the preparations studied. Thus, the results obtained confirmed the stability of phospholipid nanoparticles during freeze-drying.

### 3.2. Morphology of the Phospholipid Composition of Indomethacin Using Small-Angle Neutron Scattering

At the next stage, it was especially important to study the morphology of the Indolip composition freeze-dried after its reduction with water.

The major advantage of the small-angle neutron scattering relative to the dynamic light scattering is its ability to characterize not only the diameter of the nanoparticles, but also determine exactly the particle morphology (micelles or vesicles) [19]. Moreover, the vesicle radius, polydispersity of vesicle population, membrane thickness and internal bilayer structure can be obtained from the small-angle neutron scattering experiment [20].

The morphology of nanoparticles of the phospholipid transport nanosystem (PTNS) for drug delivery developed in the Institute of Biomedical Chemistry was investigated by means of the small-angle X-ray scattering. The results obtained allow one to answer the key question from the viewpoint of organization of drug incorporation: do the PTNS nanoparticles have a structure of micelles or vesicles? It was demonstrated that PTNS is a vesicular system with the average vesicle radius 160 ± 2 Å [21].

**Figure 3:**
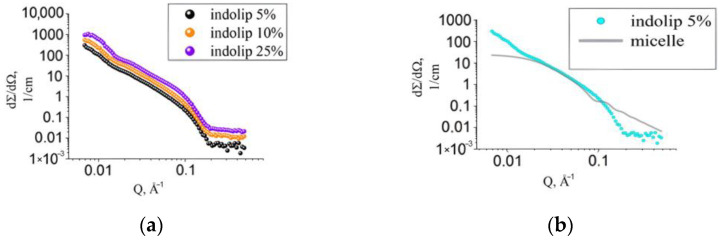
Experimental curves of small-angle neutron scattering of the Indolip preparation at various concentrations in heavy water (**a**), a sample with a concentration of 5% and the approximation function describing the micelle (**b**).

Figure 3a shows the experimental curves of small-angle neutron scattering of samples with different Indolip concentrations in heavy water: 5%, 10%, 25% (*w*/*w*).

It is important to note that at all concentrations, the ratio of phospholipid/maltose is constant and equal to ¼. After solubilization of Indolip in D_2_O, we have for the Indolip concentration of 5%:1% lipid concentration in 4% solution of the maltose in D_2_O. For the Indolip concentration of 10%:2% lipid concentration in 8% solution of the maltose in D_2_O. For the Indolip concentration of 25%:5% lipid concentration in 20% solution of the maltose in D_2_O. Concentration of the maltose in D_2_O increases with an increase in the Indolip concentration.

The composition of the Indolip lipid bilayer was obtained as a mixture of 625 mg of indomethacin and 6250 mg of soy phospholipid Lipoid S100. The ratio of the indomethacin to the phospholipid has a constant value of 1/10 in the lipid bilayer of vesicles at any concentrations of the Indolip in D_2_O.

Using the SasView program, we studied whether the Indolip belongs to micelles. The analysis showed the same result as from small-angle X-ray scattering for the phospholipid transport nanosystem [21]. The experimental curve of the studied drug Indolip could not be described by the equation corresponding to small-angle scattering from the micelles (Figure 3b).

**Figure 4:**
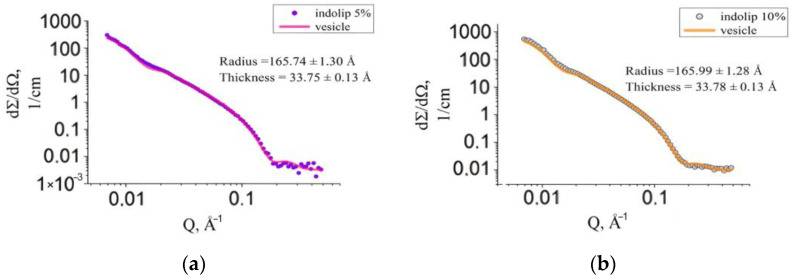
Experimental scattering curve of the Indolip sample with a concentration of 5% (**a**) and 10% (**b**) and the approximation function describing vesicles.

Calculations to determine the model of the phospholipid composition of indomethacin showed that for all concentrations of the drug studied, the experimental spectrum was well described by the hollow sphere model (vesicles). The results are shown in Figure 4.

**Figure 5:**
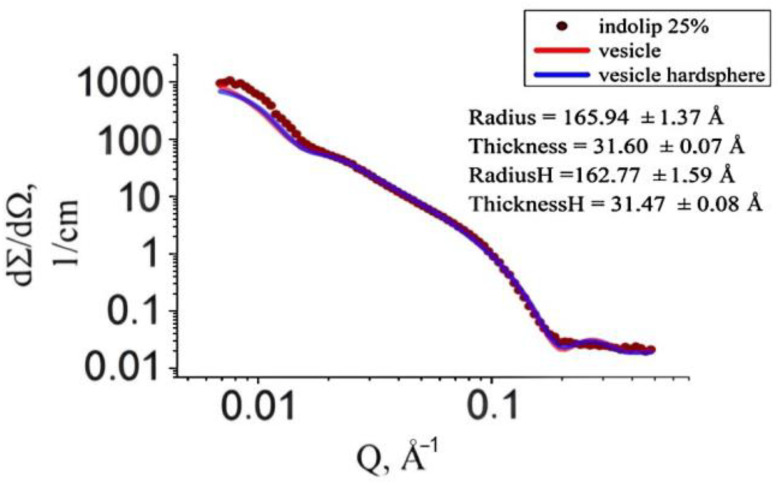
Experimental curve of small-angle neutron scattering of a sample with Indolip concentration of 25% and two approximation functions that describe vesicles taking into account the intervesicle interaction (H) and without it.

The graph in Figure 5 shows the experimental scattering curve of samples in which the concentration of the phospholipid composition of indomethacin was 25%. Figure 4 and Figure 5 show that with an increase in the concentration of the studied drug in heavy water, the vesicle function described the curve worse. When taking into account the emerging intervesicle interaction according to the model of “rigid spheres”, the approximation accuracy has not improved.

**Figure 6:**
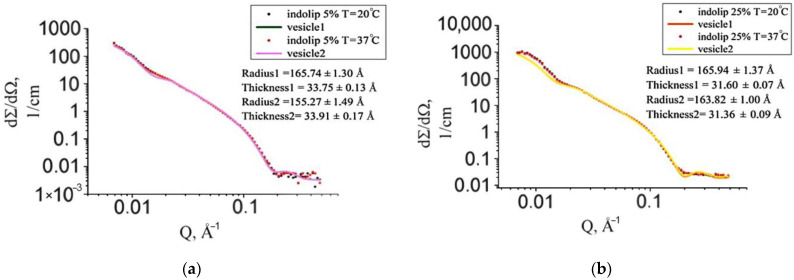
Experimental curve of small-angle neutron scattering of samples with Indolip concentration of 5% (**a**) and 25% (**b**) at 20 °C and 37 °C and their approximation functions that describe vesicles.

Since Indolip has been developed as a drug for oral administration, it was interesting to conduct research at a temperature close to human body temperature (37 °C). The experimental scattering curve of a sample with a concentration of 5% at a temperature of 20 °C practically did not differ from that of a sample with the same concentration, but at a higher temperature (Figure 6). In the case of the 25% sample, an increase in temperature also had no significant effect on the scattering curves.

Table 3 shows all the parameters obtained for the samples studied. It is worth noting that the radius is the inner radius of the vesicles. The polydispersity of the radius was described by the Schultz function, and the polydispersity of the lipid bilayer thickness was described by the Gaussian function, and the resolution function of the spectrometer was also taken into account in all approximations.

Thus, according to the data obtained, Indolip was a vesicle rather than a micelle. It was found that an increase in its concentration in heavy water from 5% to 25% did not destroy its morphology. Under conditions of human physiological temperature (37 °C) at the Indolip concentration of 25% in water, the inner radius of the vesicle was 164 ± 1 Å, and the thickness of the lipid layer was equal to 31.4 ± 0.1 Å. The radius of the vesicle exceeded the value of the bilayer thickness by five times.

The membrane thickness decreased from 33.8 ± 0.1 Å at 5% Indolip concentration 5% in D_2_O to the value of 31.5 ± 0.1 Å at 20% Indolip concentration. Indolip concentration 5% in D_2_O creates 4% solution of maltose in D_2_O and, correspondingly, Indolip concentration 20% in D_2_O creates a 16% solution of maltose in D_2_O.

The homogeneous approximation of the internal structure of the lipid bilayer used in our calculations gives an underestimated value of the membrane thickness [20]. Nevertheless, we can compare the relative changes in the thickness of lipid membranes under the influence of disaccharides.

Sucrose concentration significantly affects the vesicle structure. With an increase in the sucrose concentration in water from 0 to 40%, the bilayer thickness of dimyristoylphosphatidylcholine (DMPC) decreases to the value of 14 ± 1.6 Å [16]. Trehalose solutions in water have a similar effect. In the liquid crystalline phase, the DMPC membrane thickness decreases to a value of 3.1 Å at 20% trehalose [22].

The vesicular system was stable with changes in concentration and temperature. The ratio of radius to thickness is close to five. The concentration stability indicated the possibility of studying highly dilute systems (with Indolip concentrations in water at less than 5%). It should be emphasized that it was not possible to determine the position of indomethacin in the lipid bilayer using the homogeneous approximation applied to describe the neutron scattering length density.

## 4. Conclusions

A phospholipid transport system with indomethacin was obtained.

After lyophilization, the indomethacin composition was a freeze-dried substance in a maltose matrix, easily soluble in water. According to the analysis, in the colloidal solution obtained after dissolution, an average size of phospholipid nanoparticles with indomethacin embedded was 21.9 ± 0.9 nm. At the same time, particles with a size of 20.99 nm accounted for 95.6% and particles with a size of 177.2 nm accounted for 4.4%.

These data confirm that the phospholipid composition was a highly homogeneous nanosystem with a uniform distribution of the substance included and a predominant particle size of about 21 nm.

The average diameter of particles before and after lyophilization was approximately the same: 20.3 ± 1.3 and 21.9 ± 0.9, respectively. Light transmission (transparency) decreased slightly after lyophilization: 73.7 ± 2.3 and 65.0 ± 2.2, respectively, but its value remained above 60%.

Based on the above, lyophilization of Indolip did not lead to a change in its physicochemical parameters (particle size, light transmission). There was also no significant increase in the oxidation of phospholipids. Thus, during the lyophilization process, the properties of phospholipid nanoparticles were preserved and were similar to the properties of the drug before lyophilization.

The ζ-potential of the phospholipid transport system changed upon the inclusion of indomethacin. The ζ-potential of a drug-free sample of phospholipid nanoparticles was −3.9 ± 1.1 mV, i.e., with the inclusion of indomethacin, the absolute value of the ζ-potential increased to −12.9 ± 0.6 mV, showing an increasing stability of the phospholipid transport system containing indomethacin.

Using the method of small-angle neutron scattering, it was possible to characterize not only the size of the nanoparticles of the phospholipid transport system with indomethacin included, but also to accurately determine the morphology of the particles obtained. According to the data of small-angle neutron scattering, Indolip is a vesicle. The vesicle radius exceeded the value of the bilayer thickness by five times. The vesicular system was stable with changes in concentration and temperature.

## Figures and Tables

**Table 1:** Characteristics of indomethacin composition before and after drying.

Studied Parameter	Indomethacin in the Composition of Phospholipid Nanoparticles	
	Before Lyophilization	After Lyophilization
Diameter of phospholipid nanoparticles, nm	20.3 ± 1.3	21.9 ± 0.9
Light transmission (at 660 nm), %	73.7 ± 2.3	65.0 ± 2.2

**Table 2:** Parameters of evaluation of phospholipid oxidation in indomethacin compositions.

Studied Parameter	Indomethacin in the Composition of Phospholipid Nanoparticles		Initial PC
	Before Lyophilization	After Lyophilization	
Oxidation index	0.37 ± 0.02	0.41 ± 0.02	0.20 ± 0.013
Peroxide number, mEq/kg	3.0 ± 0.7	4.3 ± 0.8	0
lysoPC content, % by weight of phospholipids	1.9%	1.9%	1.1–1.5%

**Table 3:** Parameters of Indolip nanoparticles under various conditions.

Sample, *w*/*w* Concentration in D_2_O	5% of Indolip		10% of Indolip	25% of Indolip	25% of Indolip	
Sample temperature	20 °C	37 °C	20 °C	20 °C	20 °C	37 °C
Structural factor	-	-	-	-	Hard sphere	-
Vesicle radius, Å	166 ± 1	155 ± 2	166 ± 1	166 ± 1	163 ± 2	164 ± 1
Polydispersity of radius (Schultz distribution), %	27.6	30.0	28.0	29.0	29.0	30.0
Thickness of lipid bilayer, Å	33.8 ± 0.1	33.9 ± 0.2	33.8 ± 0.1	31.6 ± 0.1	31.5 ± 0.1	31.4 ± 0.1
Polydispersity of lipid bilayer thickness (Gaussian distribution), %	15	10	16	5	9	4
χ^2^	11.9	8.8	13.7	23.5	22.6	16.9

## Data Availability

The data is available on reasonable request from the corresponding author.
